# Supplementary material for: Retinoic Acid Sensitivity of Triple-Negative Breast Cancer Cells Characterized by Constitutive Activation of the notch1 Pathway: The Role of Rarβ
Source: Cancers (Basel). 2020 Oct 18;12(10):3027. doi: 10.3390/cancers12103027 (PMC7650753; doi:10.3390/cancers12103027)
Supplement: Supplementary file 1 [file cancers-12-03027-s001.zip › Supplementary/Suppl_Info_Cancers.pdf]

SUPPLEMENTARY INFORMATION

Title: Retinoic acid sensitivity of triple-negative breast-cancer cells characterized by constitutive activation of the NOTCH1 pathway: the role of RARβ

Gabriela Paroni[1], Adriana Zanetti[1], Maria Monica Barzago[1], Mami Kurosaki[1], Luca Guarrera[1], Maddalena Fratelli[1], Martina Troiani[1], Paolo Ubezio[2], Marco Bolis[1,3], Arianna Vallerga[1], Federica Biancardi[1], Mineko Terao[1] and Enrico Garattini[1]

[1] Laboratory of Molecular Biology, Istituto di Ricerche Farmacologiche Mario Negri IRCCS, via Mario Negri 2, 20156 Milano, Italy.

[2] Department of Oncology, Istituto di Ricerche Farmacologiche Mario Negri IRCCS, via Mario Negri 2, 20156 Milano, Italy.

[3] Functional Cancer Genomics Laboratory, Institute of Oncology Research, USI, University of Southern Switzerland; 6500 Bellinzona, Switzerland.

Supplementary Methods and References pages 2-4

Legends to Supplementary Tables S1-S2 pages 5-9

Supplementary Figures S1-S9 pages 10-18

SUPPLEMENTARY METHODS

Cell lines: The table below summarizes the characteristics and the source of the cell lines used throughout the study:

Cell name	Gender	Phenotype	Source
BT549	F	Basal (triple-negative)	ATCC
CAL-120	F	Basal (triple-negative)	DSMZ
CAL-148	F	Basal (triple-negative)	DSMZ
CAL-51	F	Basal (triple-negative)	DSMZ
CAMA1	F	Luminal (ER^+)	ATCC
HCC-1143	F	Basal (triple-negative)	ATCC
HCC-1187	F	Basal (triple-negative)	ATCC
HCC-1395	F	Basal (triple-negative)	ATCC
HCC-1419	F	Luminal ($HER2^+$)	ATCC
HCC-1569	F	Basal (triple-negative)	ATCC
HCC-1599	F	Basal (triple-negative)	ATCC
HCC-1806	F	Basal (triple-negative)	ATCC
HCC-1937	F	Basal (triple-negative)	ATCC
HCC-38	F	Basal (triple-negative)	ATCC
HCC-70	F	Basal (triple-negative)	ATCC
HDQP1	F	Basal (triple-negative)	DSMZ
Hs578T	F	Basal (triple-negative)	ATCC
MB-157	F	Basal (triple-negative)	ATCC
MCF7	F	Luminal (ER^+)	ATCC
MDA-MB-157	F	Basal (triple-negative)	ATCC
MDA-MB-175VII	F	Luminal (ER^+)	ATCC
MDA-MB-231	F	Basal (triple-negative)	ATCC
MDA-MB-361	F	Luminal (ER^+/$HER2^+$)	ATCC
MDA-MB-468	F	Basal (triple-negative)	ATCC
SK-BR-3	F	Luminal ($HER2^+$)	ATCC

ATCC = American Type Culture Collection; DSMZ = Deutsche Sammlung von Mikroorganismen und Zellkulturen. During the course of the entire study, all the cell lines were authenticated by constantly checking the morphology and the growth doubling time. In addition, all the cell lines were mycoplasma free, as indicated by periodic PCR assays performed on the cell conditioned medium, using the following nucleotide mycoplasma-recognizing primers (sense: 5'-TGCACCATCTGTCACTCTGTTAACCTC-3'; anti-sense: 5'-ACTCCTACGGGAGGCAGCAGTA-3').

All cell-lines were grown in phenol red-free Dulbecco's Modified Eagle Medium (DMEM) F12 medium (Invitrogen) supplemented with glutamine (2mM) and 5% fetal bovine serum (Lonza). Twenty four hours before treatment cells were seed in DMEM containing 5% charcoal stripped serum. In the case of ER^+ cells, estradiol (10 nM) was added to the medium.

Generation of stable cell-lines silenced for RARβ

MB-157 cells stably over-expressing RARβ-targeting shRNAs were obtained by lentiviral infection of specific constructs based on the pGreenPuro shRNA expression system (SBI, System Bioscences). The RARβ-targeting shRNAs constructs were generated by introduction of the following double-stranded oligonucleotides into the *EcoRI* and *BamHI* digested pGreenPuro vector:

shRARβ2

(sense:5'GATCCGCACAGAGAGCTATGAAATCTTCCTGTCAGAATTTCATAGCTCTCTGTGCTTTTTG3’;

antisense:5'AATTCAAAAAGCACAGAGAGCTATGAAATTCTGACAGGAAGATTTCATAGCTCTCTGTGCG3’)

shRARβ3

(sense:5'GATCCGGTAAATACACCACGAATTCTTCCTGTCAGAAATTCGTGGTGTATTTACCTTTTTG3’,

antisense:5'AATTCAAAAAGGTAAATACACCACGAATTTCTGACAGGAAGAATTCGTGGTGTATTTACCG3’).

After lentiviral infection, cells were subjected to puromycin (0.5 µg/ml) selection for the isolation of the shRARβ expressing cells.

RNA-seq studies

RNA-sequencing experiments were conducted in the *HCC-1599*, *MB-157* and *MDA-MB157* breast-cancer cell-lines. Cells were treated with vehicle (DMSO), ATRA (1 µM), DAPT (1 µM) or the ATRA+DAPT combination for 8 hours. RNA was extracted with the mRNeasy Mini Kit (QIAGEN). RNA was processed with the TruSeq stranded RNA library preparation kit (Illumina) and sequenced on the Illumina NextSeq500 apparatus with paired-end, 151-base pair long reads. The overall quality of sequencing reads was evaluated using FastQC (1).

Sequence alignments to the reference human genome (GRCh38) were performed using STAR (v.2.5.2a). Gene-expression was quantified at gene level using the comprehensive annotations made available by Gencode (2). Specifically, we used the v27 release of the Gene Transfer File (GTF). Raw-counts were further processed in the R Statistical environment and downstream differential expression analysis was performed using the DESeq2 pipeline. Genes characterized by low mean normalized counts were filtered out by the Independent Filtering feature embedded in DESeq2 (alpha = 0.05). DESeq2-computed Wald-statistics values were used as input for gene-set enrichment testing performed with the pre-ranked version of Camera (inter-gene correlation equal to 0.1, parametric test procedure). Statistical enrichments were determined for gene-sets obtained from the Hallmark collection (H), which are curated by the Molecular Signature DataBase (MSigDB) (3). All the statistical analyses were corrected for multiple comparisons, using the Benjamini-Hochberg correction method (FDR).

SUPPLEMENTARY REFERENCES

1. Andrews, S. (2010). FastQC: A Quality Control Tool for High Throughput Sequence Data [Online]. Available online at: http://www.bioinformatics.babraham.ac.uk/projects/fastqc/

2. Harrow, J., Frankish, A., Gonzalez, J. M., Tapanari, E., Diekhans, M., Kokocinski, F., Aken, B. L., Barrell, D., Zadissa, A., Searle, S., Barnes, I., Bignell, A., Boychenko, V., Hunt, T., Kay, M., Mukherjee, G., Rajan, J., Despacio-Reyes, G., Saunders, G., Steward, C., Hubbard, T. J. (2012). GENCODE: the reference human genome annotation for The ENCODE Project. Genome Research, 22(9), 1760–1774. https://doi.org/10.1101/gr.135350.111

3. Liberzon, A., Subramanian, A., Pinchback, R., Thorvaldsdóttir, H., Tamayo, P., & Mesirov, J. P. (2011). Molecular signatures database (MSigDB) 3.0. Bioinformatics (Oxford, England), 27(12), 1739–1740. https://doi.org/10.1093/bioinformatics/btr260

LEGENDS TO SUPPLEMENTARY TABLES

Supplementary Table S1 *Transcriptomic perturbations afforded by ATRA, DAPT and ATRA+DAPT in HCC-1599 and MB-157 cells*. *HCC-1599* and *MB-157* were exposed to vehicle (DMSO), ATRA (1 μM), DAPT (1 μM) and ATRA+DAPT for 8 hours. Total RNA was extracted and subjected to *RNA-seq* analysis. The various sheets contain the different analyses performed on the *RNA-seq* data as follows:

HCC1599_Expression = The expression values (CPM; Counts Per Millions) of all the mRNAs identified in *HCC-1599* cells (red columns) are shown. The blue columns show the results of the indicated comparisons performed on the expression levels of each gene. Log2FC = $\log_2$(Fold-Change); FDR = False Discovery Rate; CTRL = vehicle.

MB157_Expression = The Expression values (CPM; Counts Per Millions) of all the mRNAs identified in *MB-157* cells (red columns) are shown. The blue columns show the results of the indicated comparisons performed on the expression levels of each gene. Log2FC = $\log_2$(Fold-Change); FDR = False Discovery Rate; CTRL = vehicle.

HCC1599_ATRA_FDR = The expression values (CPM; Counts Per Millions) of all the mRNAs significantly (FDR < 0.05) up- and down-regulated by ATRA in *HCC-1599* cells (red columns) are shown. The blue columns show the results of the ATRA vs. vehicle (CTRL) comparison performed on the expression levels of each gene. Log2FC = $\log_2$(Fold-Change); FDR = False Discovery Rate.

HCC1599_DAPT_FDR = The expression values (CPM; Counts Per Millions) of all the mRNAs significantly (FDR < 0.05) up- and down-regulated by DAPT in *HCC-1599* cells (red columns) are shown. The blue columns show the results of the DAPT vs. vehicle (CTRL) comparison performed on the expression levels of each gene. Log2FC = $\log_2$(Fold-Change); FDR = False Discovery Rate.

HCC1599_ATRA+DAPT_FDR = The expression values (CPM; Counts Per Millions) of all the mRNAs significantly (FDR < 0.05) up- and down-regulated by ATRA+DAPT in *HCC-1599* cells (red columns) are shown. The blue columns show the results of the ATRA+DAPT vs. vehicle (CTRL)

comparison performed on the expression levels of each gene. Log2FC = $\log_2$(Fold-Change); FDR = False Discovery Rate.

MB157_ATRA_FDR = The expression values (CPM; Counts Per Millions) of all the mRNAs significantly (FDR < 0.05) up- and down-regulated by ATRA in *MB-157* cells (red columns) are shown. The blue columns show the results of the ATRA vs. vehicle (CTRL) comparison performed on the expression levels of each gene. Log2FC = $\log_2$(Fold-Change); FDR = False Discovery Rate.

MB157_DAPT_FDR = The expression values (CPM; Counts Per Millions) of all the mRNAs significantly (FDR < 0.05) up- and down-regulated by DAPT in *MB-157* cells (red columns) are shown. The blue columns show the results of the DAPT vs. vehicle (CTRL) comparison performed on the expression levels of each gene. Log2FC = $\log_2$(Fold-Change); FDR = False Discovery Rate.

MB157_ATRA+DAPT_FDR = The expression values (CPM; Counts Per Millions) of all the mRNAs significantly (FDR < 0.05) up- and down-regulated by ATRA+DAPT in *MB-157* cells (red columns) are shown. The blue columns show the results of the ATRA+DAPT vs. vehicle (CTRL) comparison performed on the expression levels of each gene. Log2FC = $\log_2$(Fold-Change); FDR = False Discovery Rate.

HCC1599_ATRA_FOLDCHANGE = The expression values (CPM; Counts Per Millions) of all the mRNAs significantly (FDR < 0.05 and Fold-Change > 1.4 & < 0.6) up- and down-regulated by ATRA in *HCC-1599* cells (red columns) are shown. The blue columns show the results of the ATRA vs. vehicle (CTRL) comparison performed on the expression levels of each gene. Log2FC = $\log_2$(Fold-Change); FDR = False Discovery Rate.

HCC1599_DAPT_ FOLDCHANGE = The expression values (CPM; Counts Per Millions) of all the mRNAs significantly (FDR < 0.05 and Fold-Change > 1.4 & < 0.6) up- and down-regulated by DAPT in *HCC-1599* cells (red columns) are shown. The blue columns show the results of the DAPT vs. vehicle (CTRL) comparison performed on the expression levels of each gene. Log2FC = $\log_2$(Fold-Change); FDR = False Discovery Rate.

HCC1599_ATRA+DAPT_ FOLDCHANGE = The expression values (CPM; Counts Per Millions) of all the mRNAs significantly (FDR < 0.05 and Fold-Change > 1.4 & < 0.6) up- and down-regulated by ATRA+DAPT in *HCC-1599* cells (red columns) are shown. The blue columns show the results of the ATRA+DAPT vs. vehicle (CTRL) comparison performed on the expression levels of each gene. Log2FC = $\log_2$(Fold-Change); FDR = False Discovery Rate.

MB157_ATRA_ FOLDCHANGE = Expression values (CPM; Counts Per Millions) of all the mRNAs significantly (FDR < 0.05 and Fold-Change > 1.4 & < 0.6) up- and down-regulated by ATRA in *MB-157* cells (red columns). The blue columns show the results of the ATRA vs. vehicle (CTRL) comparison performed on the expression levels of each gene. Log2FC = $\log_2$ Fold-Change; FDR = False Discovery Rate.

MB157_DAPT_ FOLDCHANGE = The expression values (CPM; Counts Per Millions) of all the mRNAs significantly (FDR < 0.05 and Fold-Change > 1.4 & < 0.6) up- and down-regulated by DAPT in *MB-157* cells (red columns) are shown. The blue columns show the results of the DAPT vs. vehicle (CTRL) comparison performed on the expression levels of each gene. Log2FC = $\log_2$(Fold-Change); FDR = False Discovery Rate.

MB157_ATRA+DAPT_ FOLDCHANGE = The expression values (CPM; Counts Per Millions) of all the mRNAs significantly (FDR < 0.05 and Fold-Change > 1.4 & < 0.6) up- and down-regulated by ATRA+DAPT in *MB-157* cells (red columns) are shown. The blue columns show the results of the ATRA+DAPT vs. vehicle (CTRL) comparison performed on the expression levels of each gene. Log2FC = $\log_2$(Fold-Change); FDR = False Discovery Rate.

HCC1599_ATRA-DAPT_Common = The expression values (CPM; Counts Per Millions) of all the mRNAs significantly (FDR < 0.05) and commonly up- and down-regulated by ATRA and DAPT in *HCC-1599* cells (red columns) are shown. The blue columns show the results of the ATRA vs. vehicle (CTRL), DAPT vs. vehicle and ATRA+DAPT vs. vehicle comparisons performed on the expression levels of each gene. Log2FC = $\log_2$(Fold-Change); FDR = False Discovery Rate.

MB157_ATRA-DAPT_Common = The expression values (CPM; Counts Per Millions) of all the mRNAs significantly (FDR < 0.05) and commonly up- and down-regulated by ATRA and DAPT in *MB-157* cells (red columns) are shown. The blue columns show the results of the ATRA vs. vehicle (CTRL), DAPT vs. vehicle and ATRA+DAPT vs. vehicle comparisons performed on the expression levels of each gene. Log2FC = $\log_2$(Fold-Change); FDR = False Discovery Rate. The genes marked in red are the genes whose expression is significantly up-regulated by ATRA+DAPT relative to ATRA alone or DAPT alone (at least $p<0.05$ following two-way ANOVA).

HCC1599_ATRA-DAPT_Common_FC = The expression values (CPM; Counts Per Millions) of all the mRNAs significantly (FDR < 0.05 and Fold-Change > 1.4 & < 0.6) and commonly up- and down-regulated by ATRA and DAPT in *HCC-1599* cells (red columns) are shown. The blue columns show the results of the ATRA vs. vehicle (CTRL), DAPT vs. vehicle and ATRA+DAPT vs. vehicle comparisons performed on the expression levels of each gene. Log2FC = $\log_2$(Fold-Change); FDR = False Discovery Rate. The genes marked in red are the genes whose expression is significantly up- or down-regulated by ATRA+DAPT relative to ATRA alone or DAPT alone (at least $p<0.05$ following two-way ANOVA).

MB157_ATRA-DAPT_Common_FC = The expression values (CPM; Counts Per Millions) of all the mRNAs significantly (FDR < 0.05 and Fold-Change > 1.4 & < 0.6) commonly up- and down-regulated by ATRA and DAPT in *MB-157* cells (red columns) are shown. The blue columns show the results of the ATRA vs. vehicle (CTRL), DAPT vs. vehicle and ATRA+DAPT vs. vehicle comparisons performed on the expression levels of each gene. Log2FC = $\log_2$(Fold-Change); FDR = False Discovery Rate.

Supplementary Table S2 *Enrichment analyses of the gene-sets up- and down-regulated by ATRA, DAPT and ATRA+DAPT in HCC-1599 and MB-157 cells* The two breast-cancer cell-lines were treated with vehicle (DMSO), DAPT (1 μM), ATRA (1 μM) and DAPT+ATRA for 8 hours. Total RNA was extracted and subjected to *RNA-seq* analysis. The gene-sets up- or down-regulated by

ATRA, DAPT and ATRA+DAPT were subjected to GSEA (Gene Set Enrichment Analysis) using the HALLMARK dataset. The FDR (False Discovery Rate) values and the ATRA-dependent up- or down-regulation are indicated. Each sheet contains the indicated comparisons.

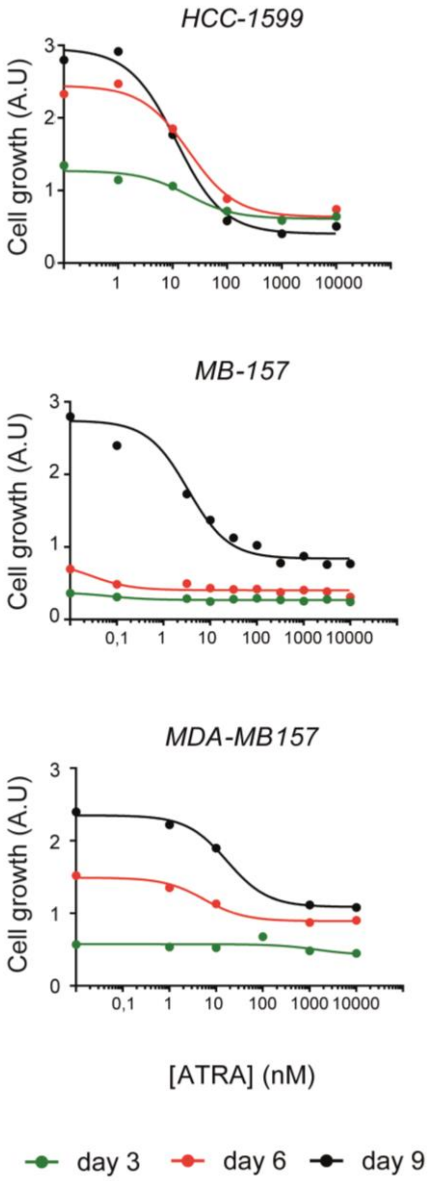

Supplementary Figure S1 *Anti-proliferative effects of ATRA in HCC-1599, MB-157 and MDA-MB157 cells*

The indicated cell lines were treated with increasing concentrations of ATRA for 3, 6 and 9 days. Sulforhodamine (*MB-157* and *MDA-MB-157* cells) and Cell Titer Glow (*HCC-1599* cells) assays were performed to determine the growth of each cell-line. The panels illustrate the dose-response curves obtained with the PRISM software. Each value is the mean of at least 6 replicate cultures.

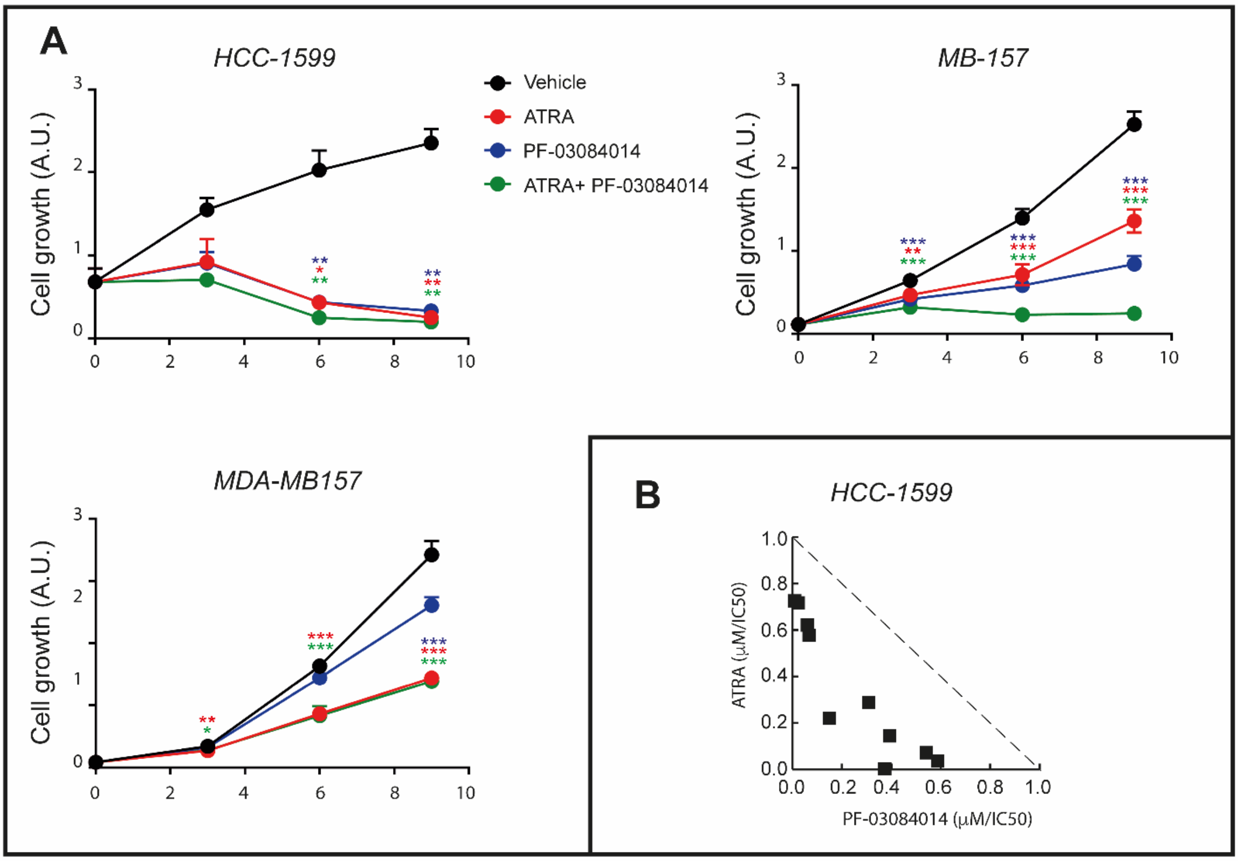

Supplementary Figure S2 *Anti-proliferative effects of ATRA, PF-03084014 and ATRA+PF-03084014 in HCC-1599, MB-157 and MDA-MB157 cells*

(**A**) The indicated cell lines were treated with ATRA (1.0 µM), PF-03084014 (0.1 µM) and the ATRA+ PF-03084014 combination for 3, 6 and 9 days, as indicated. Cell growth was determined with the Sulforhodamine assay (*MDA-MB157* and *MB-157* cells) or the CellTiter-Glo-Luminescent-Cell-Viability assay (*HCC-1599* cells). A.U. = Arbitrary Units. Each value is the mean±SD of six independent cultures. The effect of ATRA and DAPT relative to vehicle treated cells was calculated by Two-way ANOVA followed by Dunnett's test (Prism Path 8). *** Significantly different ($p<0.001$); ** Significantly different ($p<0.01$). *Significantly different ($p<0.05$). (**B**) *HCC-1599* cells were treated with vehicle (DMSO) or increasing concentrations of ATRA and PF-03084014 alone or in combination for nine days. The panel illustrates the isobologram of the data obtained with combinations of ATRA and PF-03084014. The additivity dashed line separates the antagonistic (upper) from the synergistic (lower) regions.

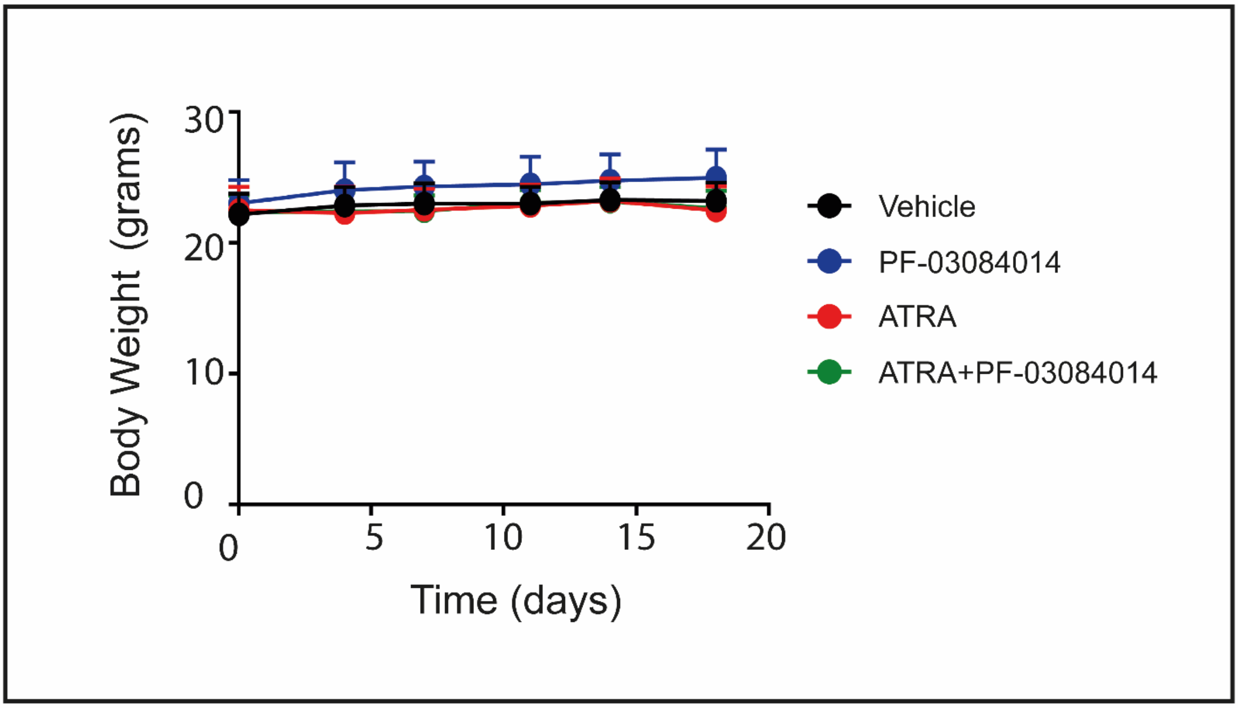

Supplementary Figure S3 *Effects of ATRA, PF-03084014 and ATRA+PF-03084014 on the body weight of mice xenotransplanted with HCC-1599 cells*

HCC-1599 cells (1×10^7/animal) were injected subcutaneously on the two flanks of female *SCID* mice. Four days after transplantation, 12 animals/experimental group were treated with: (1) vehicle; (2) PF-03084014 (90 mg/kg, *per os* twice/day) 5 days a week for a total of 18 days; (3) ATRA (15.0 mg/kg, intraperitoneally once/day) 5 days a week for a total of 18 days; (4) ATRA+PF-03084014 as in (2) and (3). The total body weight of each animal was measured at the indicated times from the start of the treatment. Each value is the Mean$\pm$S.D. of 12 animals. No significant difference among the various experimental groups was determined by Two-way ANOVA followed by Dunnett's test (Prism Path 8).

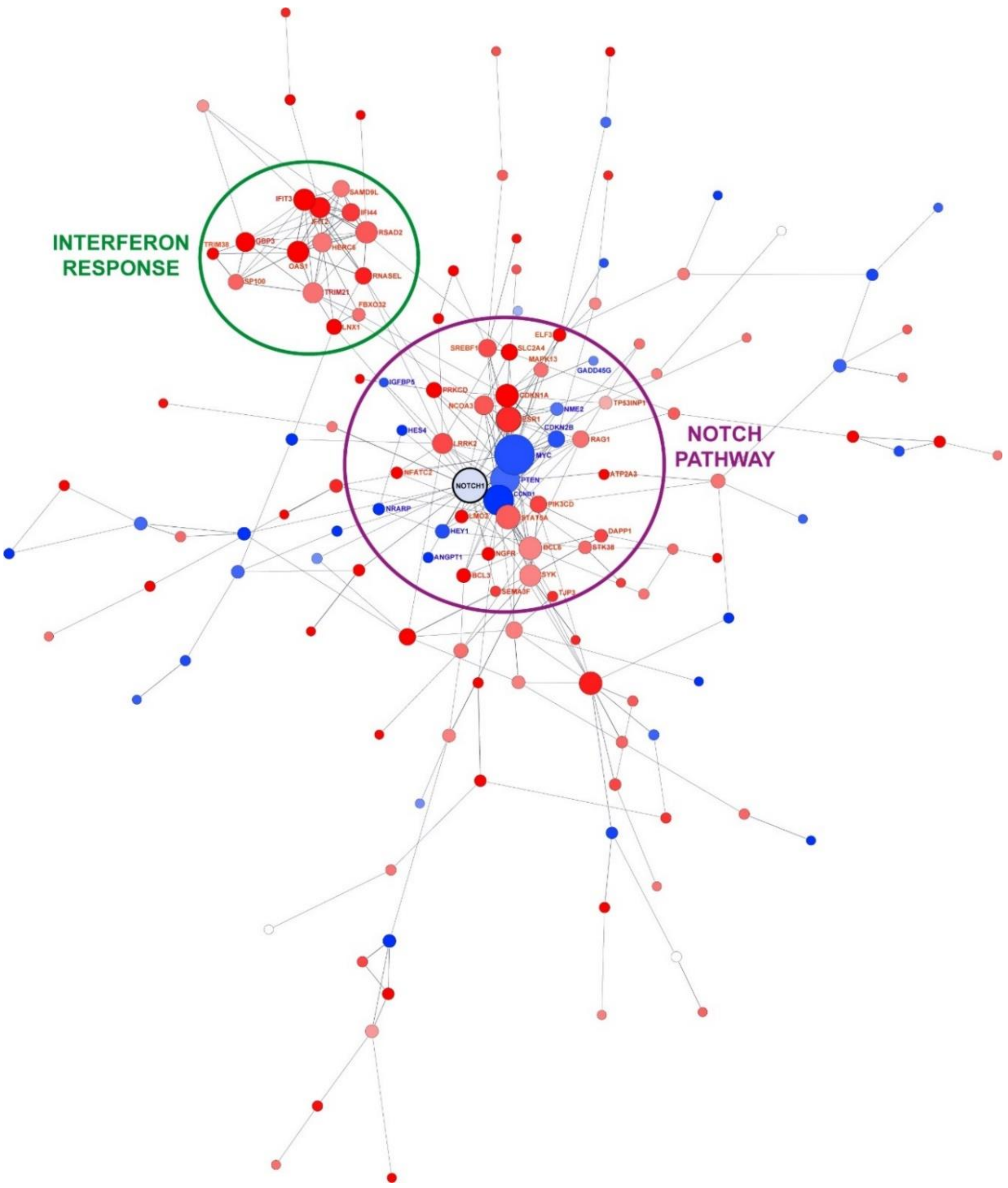

Supplementary Figure S4 *Gene-network interaction analysis of the common genes regulated by ATRA and DAPT in HCC-1599 cells*

The 220 and 60 common gene products up- and down-regulated, respectively, in HCC-1599 cells by ATRA and DAPT were subjected to a NOTCH1 oriented network interaction analysis using the publicly available Cytoscape software. The red circles indicate genes whose expression is up-regulated by ATRA and DAPT, while the blue circles indicate genes whose expression is down-regulated by the two compounds. The size of the circles is proportional to the level of up- and down-regulation observed with the combination of ATRA+DAPT. The symbols of the genes commonly up- and down-regulated by ATRA and DAPT whose products show the closest direct or indirect interactions with the NOTCH1 protein are indicated. These gene products are clustered in a group which we denominated as "NOTCH pathway" and are contained within the purple circle. A second cluster of closely interacting protein which is enriched in genes annotated as belonging to the "Interferon Response" pathway (HALLMARK gene-sets) are surrounded by a green circle and are shown as a reference. The symbols of the genes coding for this last set of proteins are also shown.

Left panel

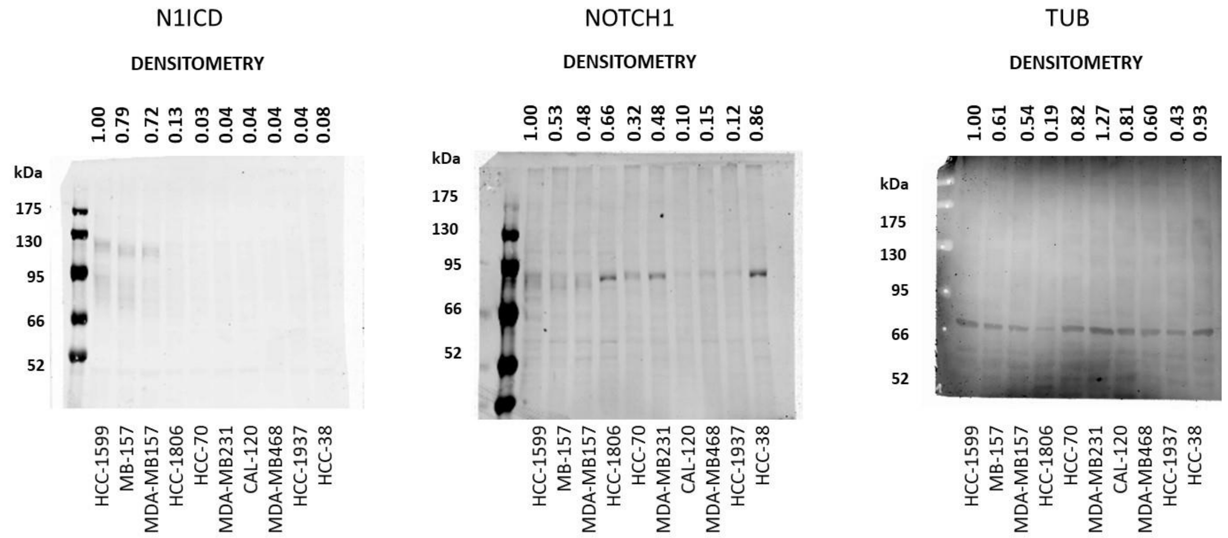

Right panel

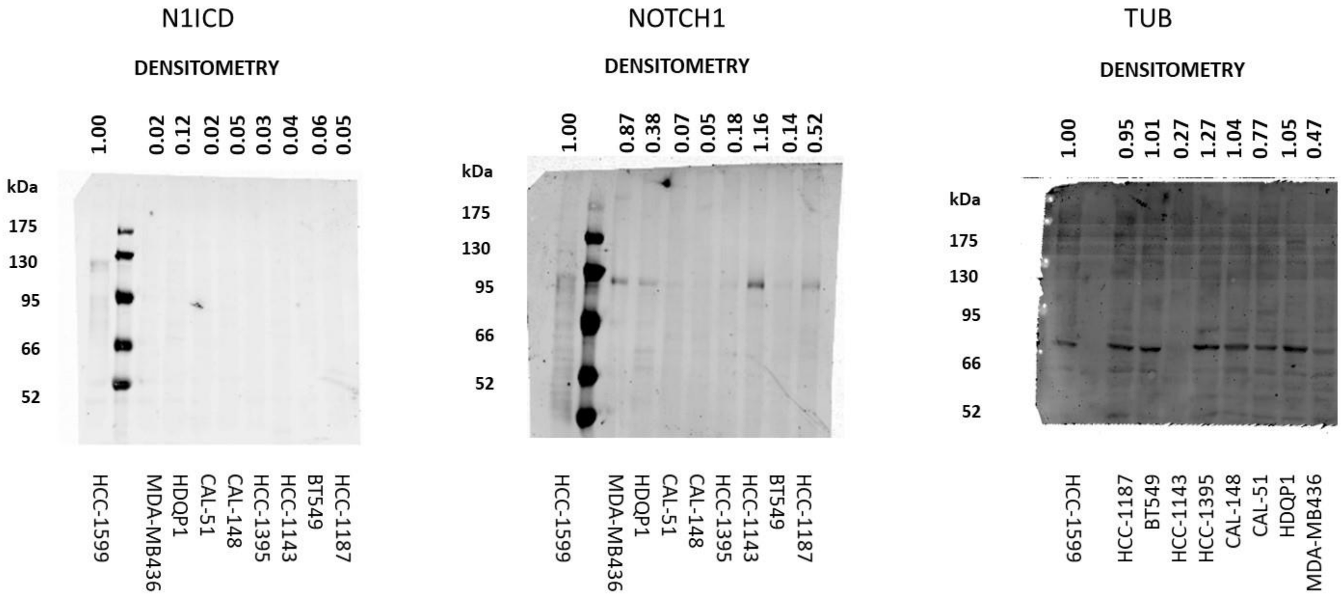

Supplementary Figure S5 *Original Western blots and densitometric analyses*
The figure shows the original Western blots contained in **Figure 1A** left and right panel, as indicated, as well as the corresponding densitometric analyses.

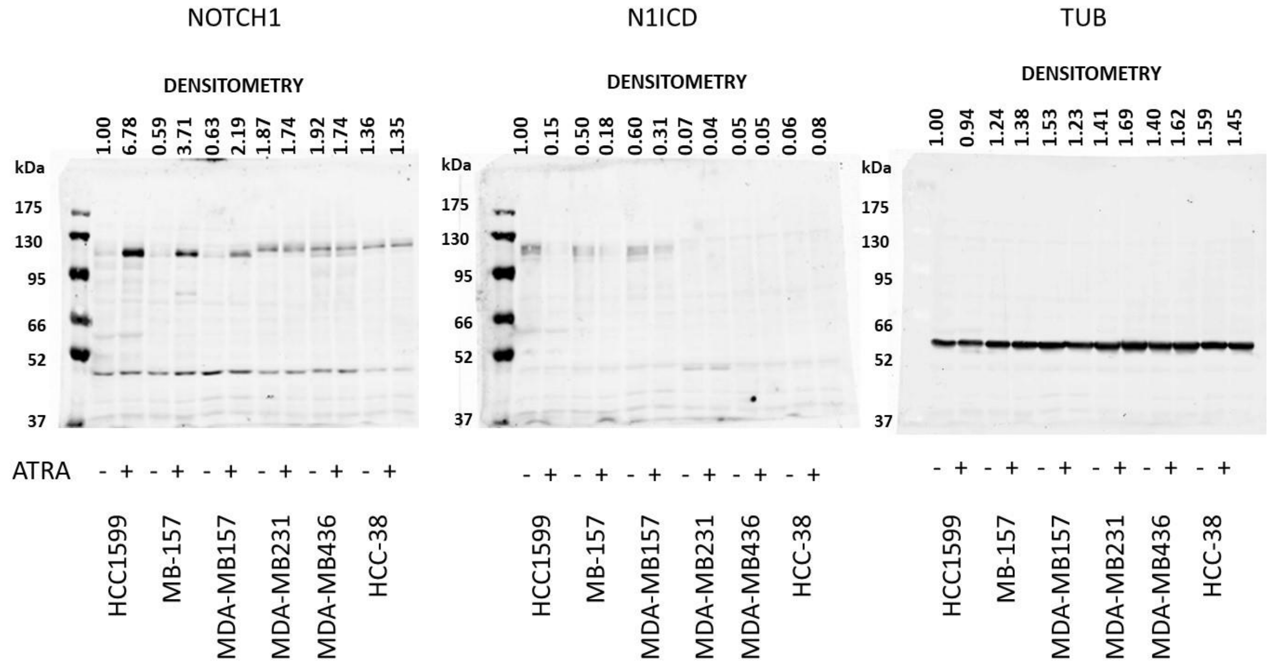

Supplementary Figure S6 *Original Western blots and densitometric analyses*
The figure shows the original Western blots contained in **Figure 2B** and the corresponding densitometric analyses.

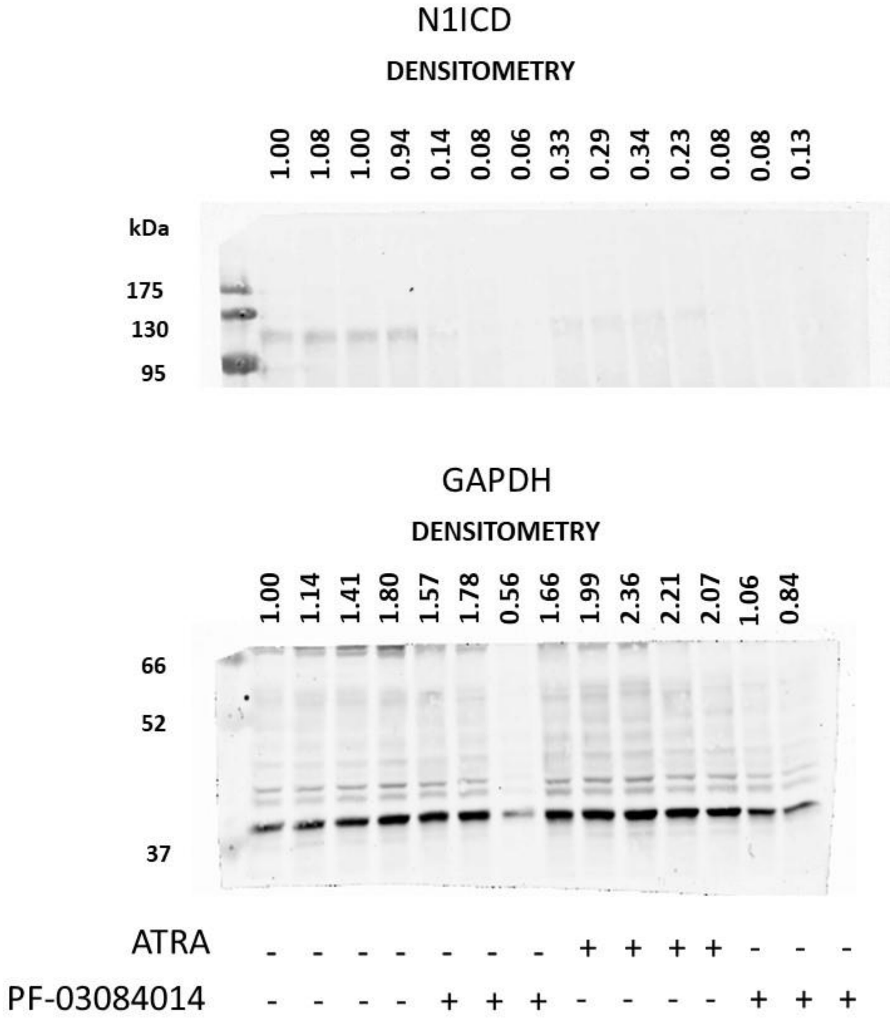

Supplementary Figure S7 *Original Western blots and densitometric analyses*
The figure shows the original Western blots contained in **Figure 4D** and the corresponding densitometric analyses.

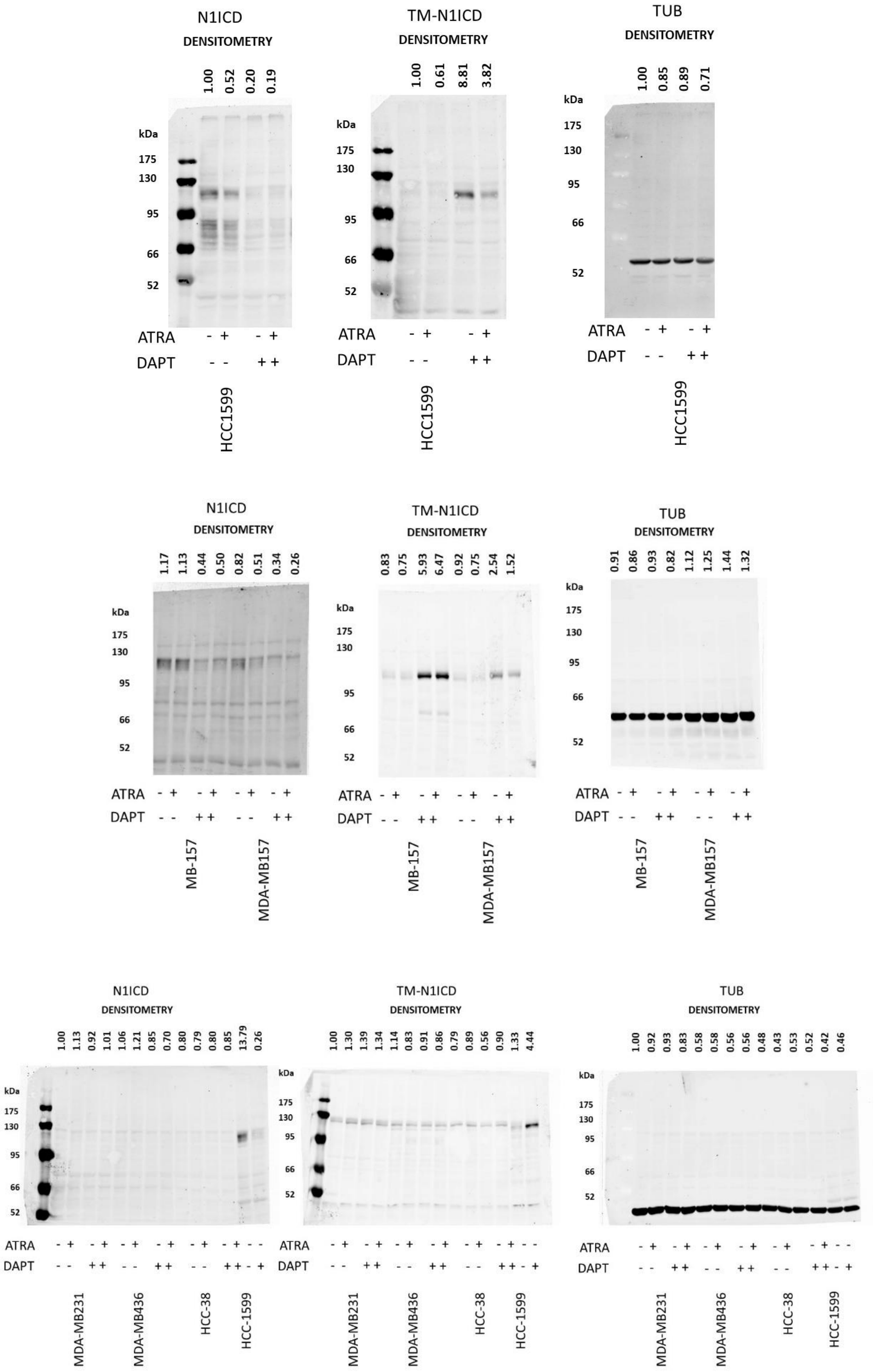

Supplementary Figure S8 *Original Western blots and densitometric analyses*
The figure shows the original Western blots contained in **Figures 5A** (upper and middle 3 panels) and **5C** (lower 3 panels) as well as the corresponding densitometric analyses.

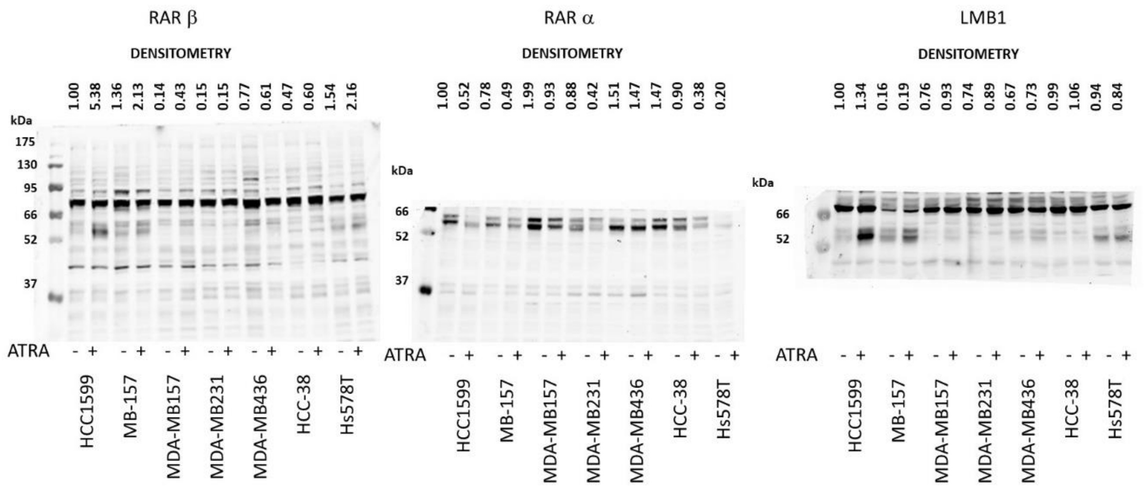

Left panel

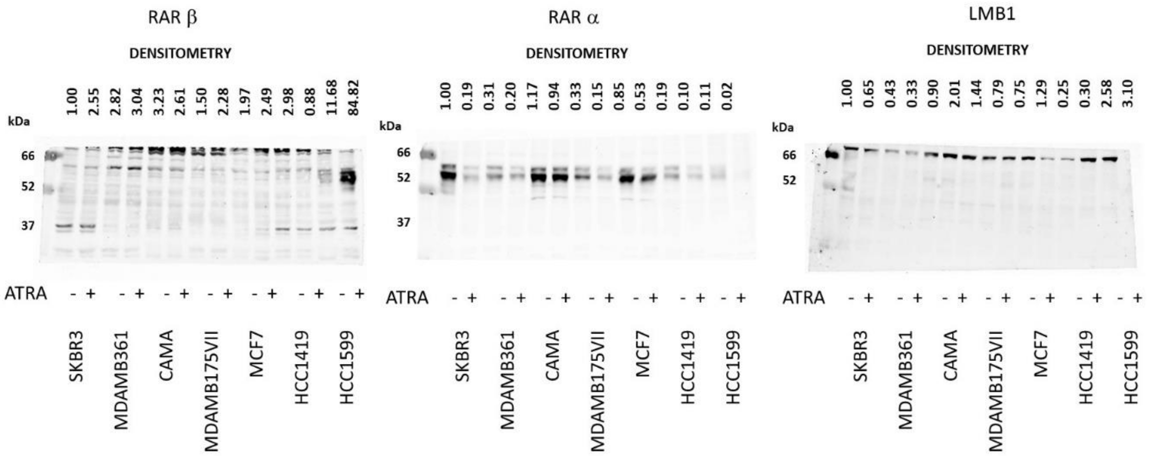

Right panel

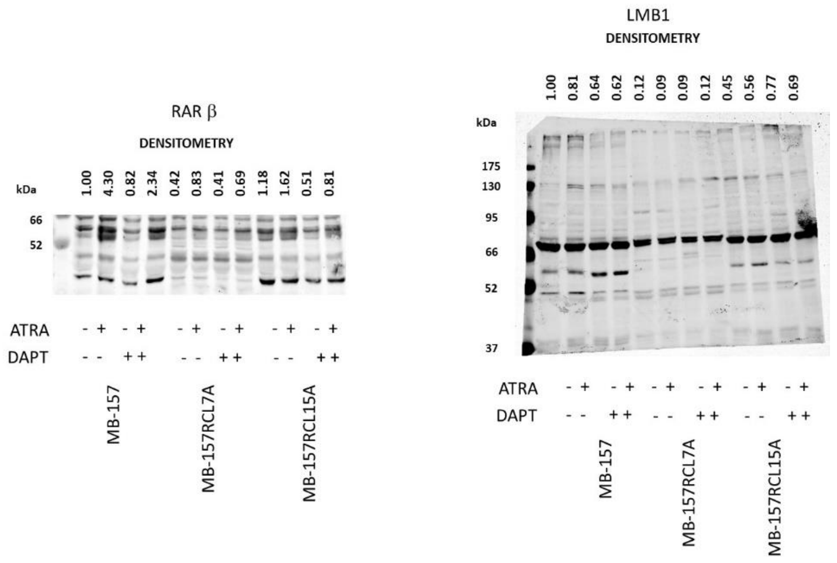

Supplementary Figure S9 *Original Western blots and densitometric analyses*

The figure shows the original Western blots contained in **Figures 8B** left and right panel (upper and middle 3 panels, as indicated) and **8C** (lower 2 panels) as well as the corresponding densitometric analyses.

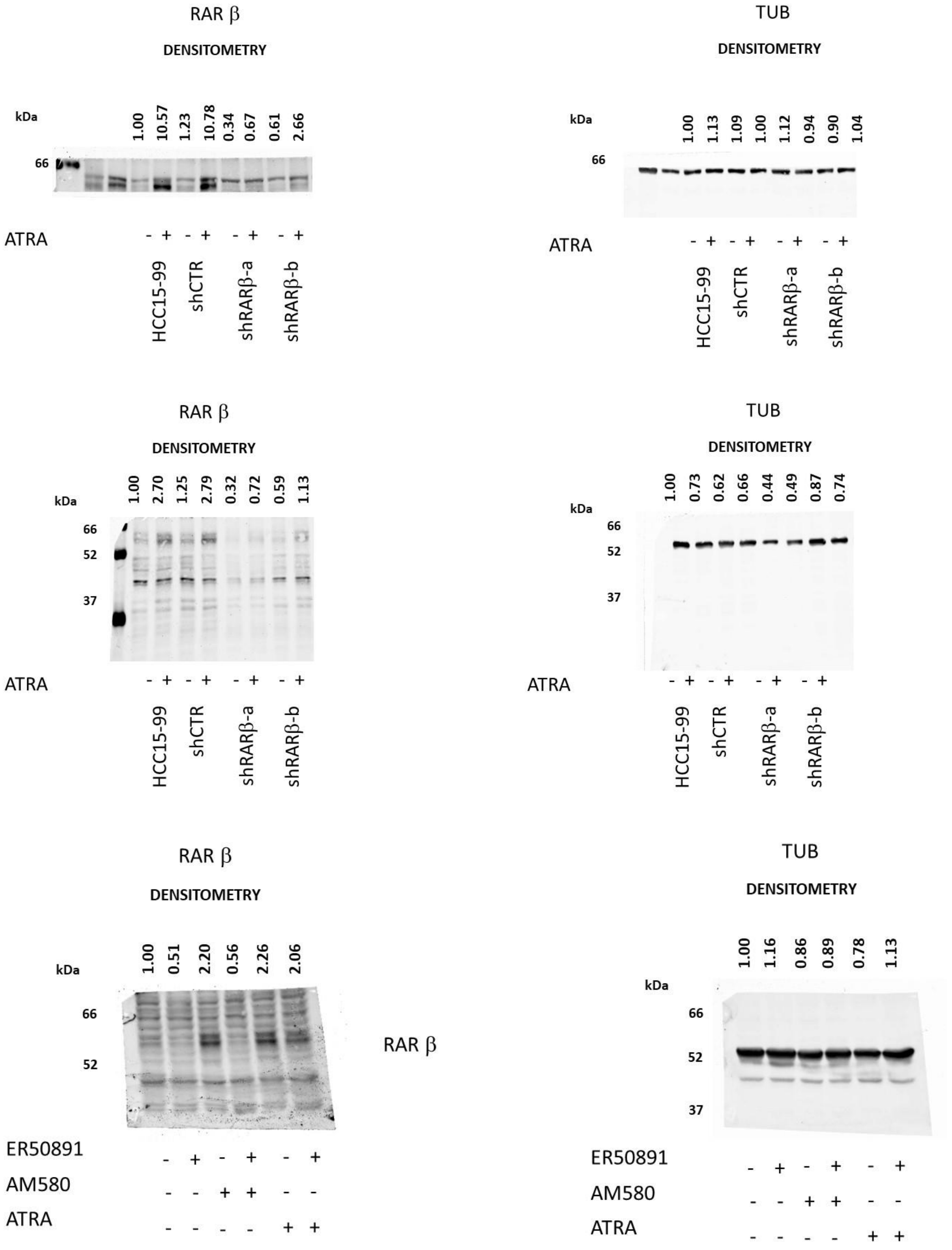

Supplementary Figure S10 *Original Western blots and densitometric analyses*
The figure shows the original Western blots contained in **Figures 8D** (upper 2 panels), **8E** (middle 2 panels) and **8F** (lower 2 panels) as well as the corresponding densitometric analyses.
